# Female Authorship Representation in *Arquivos Brasileiros de Oftalmologia* throughout its 80 years of existence

**DOI:** 10.5935/0004-2749.202200101

**Published:** 2022

**Authors:** Giovana Rosa Gameiro, Gustavo Rosa Gameiro, Camila V. Ventura, Paulo Schor

**Affiliations:** 1 Faculdade de Medicina, Universidade Estadual de Londrina, Londrina, PR, Brazil; 2 Department of Ophthalmology and Visual Sciences, Escola Paulista de Medicina, Universidade Federal de São Paulo, São Paulo, SP, Brazil; 3 Health Education, Centro de Desenvolvimento de Educação Médica, Faculdade de Medicina, Universidade de São Paulo, São Paulo, SP, Brazil; 4 Department of Ophthalmology, Fundação Altino Ventura, Recife, PE, Brazil; 5 Department of Ophthalmology, Hospital de Olhos de Pernambuco, Recife, PE, Brazil

It was not until 1849 that Elizabeth Blackwell became the first woman to be granted a medical degree in the United States, and only half a century later, women started entering medical or surgical specialization programs^(1)^. The first two women to establish a female hallmark in ophthalmology were Drs. Isabel Barrows and Elizabeth Sargent, who trained in the late 19^th^ century in the United States^(1)^.

Although ophthalmology ranks third among the surgical specialties in female representation, it has been a long journey for women to conquer space and gain respect among their peers in ophthalmology^(2)^. Despite all progress, studies show that women remain underrepresented in academic medicine and research^(3,4)^. Given that research productivity is vital to career progress in the academic world, identifying gender differences and gaps in scientific articles has become necessary for a better understanding of inequalities among men and women^(3,4)^.

Authorship analysis allows the understanding of the academic status of women in the hierarchical scientific system. It is of common practice that the first authorship position in original articles is occupied by early-career researchers whose efforts underlie the entire paper^(5)^. However, the last authorship in the manuscript is also considered a prestigious position that indicates the person whose work or role made the study possible and is usually occupied by senior researchers^(5)^.

Authorship trends have already been studied in several medical field journals, including *JAMA, The Lancet, and The New England Journal of Medicine*^(6-8)^. It has also been investigated in different ophthalmology journals, including *JAMA Ophthalmology, Ophthalmology,* and *Journal of Glaucoma,* and several pediatric and strabismus journals^(4,9-12)^. These previous publications have described the persistence of the gender gap in science^(4,6-12)^.

To better understand the trend of Brazilian female ophthalmologists in academic medicine and research, we performed an analysis of the first and last authorship of all articles published in the journal *Arquivos Brasileiros de Oftalmologia (ABO)*-a traditional and peer-reviewed ophthalmology journal in Brazil.

This is a retrospective study that used secondary data that are easily available on online scientific databases. The investigators consulted a previous editor-in-chief of the *ABO* and as all information analyzed in this study is open access and the study did not involve examination or treatment of patients or a review of medical records, a review by research ethics committees was waived. Names of both the first and last authors of the following online available articles of the *ABO* were included in the analysis and classified by sex: first ten years of existence of the journal (1938, 1939, 1940, 1941, 1942, 1943, 1944, 1945, 1946, and 1947), from 1950-2005 in intervals of 5 years (1950, 1955, 1960, 1965, 1970, 1975, 1980, 1985, 1990, 1995, 2000, and 2005) and the last decade of publication (2010, 2011, 2012, 2013, 2014, 2015, 2016, 2017, 2018, and 2019).

The online platform Gender API (Munich, Germany, available at https://gender-api.com/) was used to help in the determination of the sex of the authors. In cases when a returning score of <90% confidence was obtained, the investigators manually performed an extensive Google Search (Google Inc., Mountain View, CA) to identify the sex of the authors. Publications with a single author were allocated into the first author cohort. The types of publications were divided into three categories: editorial, original research, and others (e.g., letters, review articles, case reports, and clinical updates).

Linear regressions were used to explore tendencies of the data, and chi-square tests were used to compare count proportions. Statistical analyses were performed using appropriate statistical methods with IBM 24.0 SPSS^®^ software (Chicago, IL, USA) and GraphPad Prism version 9.1 for Mac (GraphPad software, San Diego, CA, USA). A p-value<0.05 was considered significant.

Of the 1833 articles published in the *ABO* since its establishment, 1801 (98.2%) were included in this study. The sex of the authors of 32 articles was not identifiable; therefore, these articles were excluded from our analysis.

In the first decade of publication (1938-1947), 165 articles were analyzed. Of these, 153 were written by single authors; none were women. When analyzing the multiple-authorship articles in that same period, there was no female first authorship and one (8.3%) female last authorship in an original article. Furthermore, considering the 53 publications of the years 1950, 1955, 1960, and 1965, there was only one article with female first authorship (1.9%).

**Figure 1:**
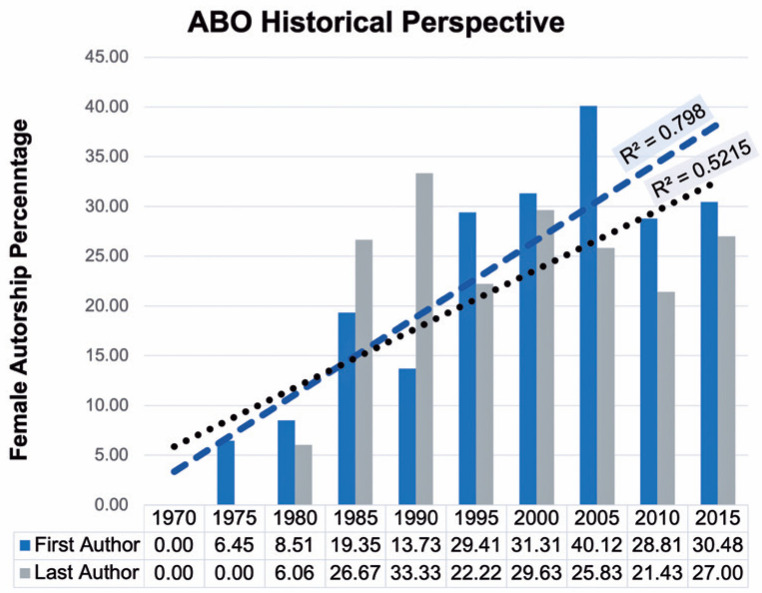
Graphic illustrating the percentage of female first and last authorship tendency since 1970, after introduction of the peer review process

Table 1 summarizes the evolution of female first and last authorship in papers published in the ABO in intervals of 5 years since the 1970s, when systematic peer review was implemented in the ABO^(13)^. Figure 1 shows that there was a significant increasing trend in the percentage of both female first authorship (slope=0.776 [0.458, 1.095] and =0.798) and last authorship (slope=0.593 [0.130, 1.057] and = 0.521) during this period (p=0.0005 and p=0.0183, respectively).

**Table 1:** Female authorship evolution of *Arquivos Brasileiros de Oftalmologia* (*ABO*) since peer review implementation

			1970	1975	1980	1985	1990	1995	2000	2005	2010	2015	Slope [Cl], R^2^, p-value
N		Editorials		3	1	3	4	1	8	12	7	6	
		Original	11	23	45	28	47	63	55	102	71	60	
		Others	0	5	1	0	0	21	36	58	40	39	
		**Total**	1 1	**31**	**47**	**31**	**51**	**85**	**99**	**172**	**118**	105	**2.810 [1.433,** **4.1861,0.735, p = 0.0015**
N		Multiple Authors	3	11	33	15	42	72	81	151	112	100	
		Single Author	8	20	14	16	9	13	18	21	6	5	
N (%)		Female Single	0 (0.00)	1 (5.00)	0 (0.00)	2 (12.50)	0 (0.00)	1 (7.70)	2 (11.11)	3 (14.28)	0 (0.00)	0 (0.00)	**0.048 [-0.266, 0.3631, 0.015, p = 0.7319**
First Author Female - N (%)		Editorials		0 (0.00)	0 (0.00)	1 (33.33)	0 (0.00)	**0 (0.00)**	**0 (0.00)**	1 (8.33)	**0 (0.00)**	1 (16.67)	
		Original	0 (0.00)	2 (8.70)	4 (8.89)	5(17.86)	7(14.89)	20 (31.75)	22 (40.00)	48 (47.06)	26 (36.62)	20 (33.33)	
		Others		0 (0.00)	0 (0.00)			5 (23.81)	9 (25.00)	20 (34.48)	8 (20.00)	11 (28.20)	
		**Total**	**0 (0.00)**	**2 (6.45)**	**4 (8.51)**	**6 (19.35)**	**7 (13.73)**	**25 (29.41)**	**31 (31.31)**	**69 (40.12)**	**34 (28.81)**	**32 (30.48)**	**0.776 [0.458,** **1.0951, 0.798, p = 0.0005**
Last Author Female - N (%)		Editorials		0 (0.00)	0 (0.00)		0 (0.00)	**0 (0.00)**	0 (0.00)	**0 (0.00)**	0 (0.00)	0 (0.00)	
		Original	0 (0.00)	0 (0.00)	2 (6.06)	4 (26.67)	14(33.33)	14(23.33)	14(25.45)	23 (23.47)	18 (25.35)	20 (33.33)	
		Others		0 (0.00)	0 (0.00)			2 (18.18)	10(43.48)	16(32.00)	6(18.75)	7(19.44)	
		**Total**	**0 (0.00)**	**0 (0.00)**	**2 (6.06)**	**4 (26.67)**	**14 (33.33)**	**16 (22.22)**	**24 (29.63)**	**39 (25.83)**	**24 (21.43)**	**27 (27.00)**	**0.593 [0.130,** **1.057], 0.521, p = 0.0183**

Absolute article number and the correspondent percentage inside the parenthesis. Linear regression was calculated: last column shows the slope with the 95% confidence interval, R^2^ of the fitting line, and p-value of the slope significantly non-zero.

The table in Figure 1 presents percentages of female authorship. Lines were calculated using the linear regression model.

**Figure 2:**
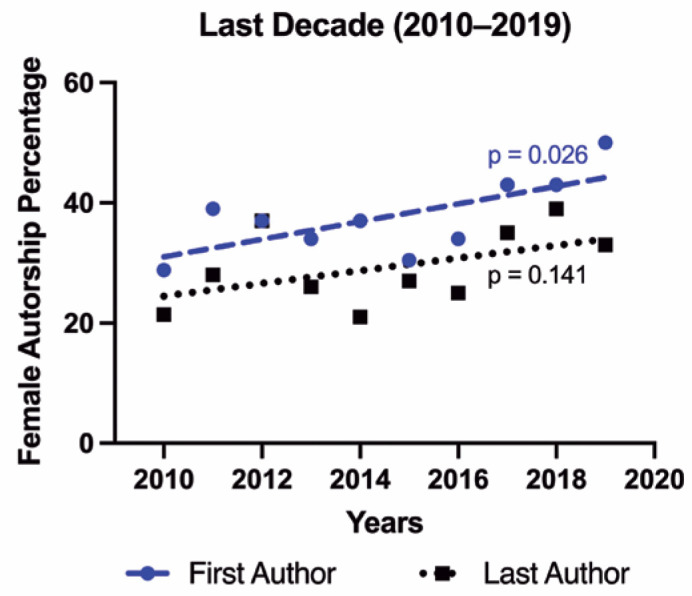
Regression lines illustrating the percentage of female first and last authorship tendency in the last decade. P-value of slope significantly non-zero.

During the last decade (2010-2019), the rise in female first authorship remained significant (p=0.026); however, this tendency was not observed in female last authorship (p=0.141), with respective slopes of 95% confidence interval of 1.468 (0.228, 2.708) and 1.055 (-0.433, 2.543). Table 2 and figure 2 show a comprehensive data review of this period.

**Table 2:** Female authorship evolution in the *ABO* in the last decade

			2010	2011	2012	2013	2014	2015	2016	2017	2018	2019	Slope [Cl], R^2^, p-value
First author Female - N (%)		Editorials	0 (0.00)	0 (0.00)	0 (0.00)	0 (0.00)	2 (28.57)	1 (16.67)	0 (0.00)	1 (14.28)	**0 (0.00)**	3 (50.00)	
		Original	26 (36.62)	26 (43.33)	24 (40.68)	19 (32.20)	23 (38.33)	20 (33.33)	19 (31.67)	30 (50.00)	33 (55.93)	25 (43.86)	
		Others	8 (20.00)	15 (37.50)	12 (37.50)	16 (41.02)	13(37.14)	11 (28.20)	22 (42.31)	12 (36.36)	11 (29.73)	23 (57.50)	
	Fema	**Total**	**34 (28.81)**	**41 (38.68)**	**36 (37.11)**	**35 (33.98)**	**38 (37.25)**	**32 (30.48)**	**41 (34.45)**	**43 (43.00)**	**44 (42.72)**	**51 (49.51)**	**1.468 [0.228,** **2.708], 0.482, p = 0.026**
Last author Female - N (%)		Editorials	0 (0.00)	0 (0.00)	0 (0.00)	0 (0.00)	1 (25.00)	0 (0.00)	2 (33.33)	0 (0.00)	1 (33.33)	0 (0.00)	
		Original	18 (25.35)	16(27.12)	25 (42.37)	18(30.51)	9(15.52)	20 (33.33)	16 (27.12)	23 (38.33)	26 (44.07)	19(33.33)	
		Others	6(18.75)	12 (33.33)	9(31.03)	6(17.65)	10(28.57)	7(19.44)	11 (22.00)	11 (34.37)	10(30.30)	13 (38.23)	
		**Total**	**24 (21.43)**	**28 (28.28)**	**34 (37.36)**	**24 (25.53)**	**20 (20.62)**	**27 (27.00)**	**29 (25.22)**	**34 (35.05)**	**37 (38.95)**	**32 (33.33)**	**1.055 [-0.433, 2.543], 0.251, p = 0.141**

Absolute article number and the correspondent percentage inside the parenthesis. Linear regression was calculated: last column shows the slope with the 95% confidence interval, R^2^ of the fitting line, and p-value of slope significantly non-zero.

Table 3 shows the Brazilian Medical Demography data from five studies^(14-18)^, which depict female representation in ophthalmology in Brazil from 2011-2020. Similarly, these studies reveal a recent increase in the number of women in ophthalmology in Brazil: slope [95% CI]=0.353 [0.074, 0.631] (p=0.027).

**Table 3:** Percentage of women in ophthalmology in Brazil according to the Brazilian Medical Demography study from the Brazilian Federal Council of Medicine (CFM) and the University of São Paulo Medical School^(14-18)^

Brazilian Medical Demography		2011	2013	2015	2018	2020	Slope [CI], R^2^, p-value
N	**Total**	9.278	9.853	11.763	13.825	15.523	
Female	**N**	3.450	3.616	4.389	5.420	6.194	**0.353 [0.074, 0.631], 0.844, p = 0.027**
	**%**	37.2	36.7	37.3	39.2	39.9	

Linear regression was calculated: last column shows the slope with the 95% confidence interval, R^2^ of the fitting line, and p-value of slope significantly non-zero.

Table 4 presents a mismatch analysis between the first and last authors’ sex in publications in the ABO from 2010-2019. A total of 996 articles with more than one author were included. The analysis shows an agreement between the first and last authors being of the same sex.

**Table 4:** Analysis of sex between the first and last authors in articles in the ABO from 2010-2019

Chi-square <0.001			Last author		Total
			Female	Male	
**First author**	**Female**	Count	141	253	394
		% Table	14.2	25.4	39.6
		% Column	48.8	35.8	
		Remainder	3.8%	-3.8%	
	**Male**	Count	148	454	602
		% Table	14.9	45.6	60.4
		% Column	51.2	64.2	
		Remainder	-3.8%	3.8%	
**Total**		Count	289	707	996
		% Table	29.0	71.0	100.0

Female representation in the medical field had a considerable increase in the last century in Brazil. In 1960, women accounted for only 13% of the physicians in the country. In 2020, this percentage increased to 46.6%^(18)^. Moreover, among younger generations, women represent most of the medical workforce- 58.5% of the physicians aged up to 30 years and 55.3% of the physicians aged between 30 and 34 years are female. This increasing trend in female representation can also be observed in the field of ophthalmology. In 2020, 39.9% of the ophthalmologists in Brazil were women, compared to 37.2% in 2011^(18)^.

The growth of female representation in the medical field is also reflected on the academic careers, as previous studies have demonstrated a significant increase in the percentage of female authors over time in original ophthalmology publications. However, it is noteworthy that this increase is more evident in first authorships than in last authorships^(9,10)^, which corroborates with the results found in this study.

Traditionally, the position of first author is reserved to the person who had the greatest participation in planning, performing the study, and writing the manuscript. The prestigious last authorship is usually reserved to the senior investigator, who plays a crucial role supporting the study both intellectually and financially^(11,19)^. As first authorship usually represents early-career scholars, such as residents, fellows, and junior faculty members, our findings may indicate that, with the recent increase in the number of young female ophthalmologists, the number of women at the junior academic level is also increasing, reproducing this trend. However, since this increase in women’s representation in medicine has been observed mainly in the last couple of decades^(18)^, probably there has been inadequate time for these junior level first authors to ascend to senior author positions^(20)^, and this could partially explain the different growth rates of women first and last authors evidenced in this study.

Although “career time” may explain the difference of slope steepness between female representation as a first author versus last author, it seems that the lower prevalence of female last authorship is a multifactorial issue. As an example, female representation in leadership roles in ophthalmology does not show significant changes over time^(6,9,21,22)^. Leadership appointments play an important role in the academia, increasing one’s reputation and visibility, which leads to career advancement^(23,24)^. They are echoed in academic and social recognition, high impact publications, invited lectures, conference presentations, and media exposure^(23,24)^. Recent publications indicate that women remain underrepresented in senior leadership positions in the field^(9,10,21,22)^.

Moreover, it is widely known that research and publications are strong credentials that can lead to academic promotion^(25)^. Conversely, academic promotion leads to a wide variety of research and academic opportunities, which further facilitate one’s career progression^(23)^. Thus, authorship positions may even help perpetuate the vicious cycle of sex disparity: the more one gets published, the more opportunities for progression as recognized and accomplished physician-scientists come their way, making it more likely for them to get published again in the future. However, the less women get published, especially in prestigious positions such as first and last authors, the less academic opportunities and visibility they will have. This scenario also contributes to fewer women in leadership positions and, as a result, increases their difficulties climbing the career ladder.

Being invited to author an editorial reaffirms one’s authority, visibility, and expertise in the field^(23)^. The present study found that editorials published in the ABO over time were predominantly written by men (Table 1), which corroborates with Franco-Cardenas et al.^(11)^. Moreover, as of today, the ABO has never had a woman in the position of editor-in-chief. Serving as an editorial board member of a renowned journal is a prestigious appointment that denotes one’s significant academic contributions and achievements in the given area^(21)^. Lastly, all current administrative board members and 62.5% of the associate editors are male. These findings reflect the underrepresentation of women as editors of ophthalmology journals^(21-23)^.

Despite the lack of data from Brazil, a study from 2018 found that ophthalmology department chairs remain predominantly male in the United States^(26)^. Another reflection of sex disparity can be observed in wage differences. A study recently conducted in Brazil reported that the sex pay gap between male and female physicians persisted even after adjusting for variables such as weekly workload, number of on-call shifts, length of practice, and specialization. The same study also showed that women are underrepresented in higher paying positions^(27)^.

Authorship sex associations may be involved in the persistence of sex disparities in ophthalmology journals. Previous studies already demonstrated that authors are more likely to work jointly with people of the same sex^(9,20)^. The current study, as shown in table 4, supports this idea, showing that 75.4% of the publications in the *ABO* with a male first author also have a male last author. However, only 35.8% of the female first authors had a female last author, which could be partially explained due to the overall higher prevalence of male last authors. These findings are important as last authors usually have a mentor-mentee relationship with first authors^(20)^.

Identifying the issues associated with the underrepresentation of women in senior-level positions and, most importantly, making efforts to support women’s career progression in the academia are crucial measures to decrease sex disparity in the field^(20,24)^. Despite the increase in the number of graduating female physicians and ophthalmologists in Brazil, women seem to fail to advance appropriately to senior ranks or they simply choose not to pursue an academic career in ophthalmology^(9)^. Studies show that women usually take on most of the household and familial duties and are generally responsible for child-rearing^(21,24)^. For these reasons, they tend to seek a flexible working schedule and maternity leave, which may contribute to the slow growth rate of women in senior academic and leadership positions^(21,24)^.

The reasons behind the sex authorship disparity highlighted by this study appear to be diverse and complex. Institutional barriers, lack of mentorship, absence of support systems, societal constraints, and even unconscious biases may also play a part in the sex differences among male and female authors^(9)^. Thus, the implementation and expansion of women mentorship programs in ophthalmology may have a positive impact by putting them in touch with potential role models and inspiring them to pursue an academic career and, consequently, leadership positions^(20)^.

An initial action for promoting sex equality in the academic field of Brazilian ophthalmology is tracking female trend in authorship and leadership roles. For instance, a real-time online platform that monitors academic journals sex authorship statistics is a feasible way of displaying sex gaps in the scientific community that could effectively direct efforts toward mitigating them. Another possible solution would be to orientate journals to seek for balance between men and women when selecting peer reviewers. This could have an impact regarding the sex gap in the authorship of editorials, as reviewers are commonly asked to write editorials^(23)^. Encouraging sex-blind collaborations^(20)^ and developing a data-driven, objective tool to determine qualified candidates for senior leadership positions, invited authorship appointments, and speaker roles could also represent a non-biased and merit-based recruitment process^(24)^.

One of the limitations of the present study is that a single ophthalmology Brazilian journal was analyzed. However, the ABO is the peer-reviewed ophthalmology journal with the highest impact factor in Brazil, and the only one indexed in the Web of Science. Second, we used a previously validated online tool for sex identification, which was not capable of identifying the sex of a minority of authors. Notwithstanding, the unidentifiable articles were excluded from the analyses, and we performed manual confirmations of the authors’ sex, if necessary, for those that were included. In a similar way, the ≥90% cutoff confidence level that was used in the online platform results was arbitrary, and some women authors may have traditionally male names and vice versa. Additionally, we assumed, based on tradition, that the last author of the publications was the principal investigator or senior member of the research team. Nevertheless, there is no official norm in the literature regarding author order^(11)^. Lastly, we analyzed a Brazilian ophthalmology journal but did not exclude international authors, which may have introduced additional bias to our findings, as different locations may have different sex distributions among ophthalmologists.

In conclusion, this editorial shows that there was an increase in female first and last authorship in the ABO throughout the analyzed period. Nevertheless, in the last decade, a significant increase was noted only in female first authorship, while female last authorship remained at low levels compared to their male colleagues. These findings reflect the persistence of women’s underrepresentation in academic medicine, particularly among senior positions. The reasons behind it deserve further investigation and should be addressed in future studies. Women’s contributions to research in ophthalmology must be stimulated and celebrated. Real scientific progress is only obtained through the debate of new ideas and perspectives and deeply relies on inclusion and diversity.
